# The Implementation and Role of Antigen Rapid Test for COVID-19 in Hemodialysis Units

**DOI:** 10.3390/ijerph192215319

**Published:** 2022-11-19

**Authors:** Jing Qi, Jia Neng Tan, Soh Heng Hui, Neoh Choo Lim, Titus Lau, Sabrina Haroon

**Affiliations:** Division of Nephrology, National University Hospital, Singapore 119074, Singapore

**Keywords:** COVID-19, hemodialysis unit, antigen rapid testing

## Abstract

As we move into the third year with COVID-19, many countries have attempted to manage the disease as an endemic. However, this is limited by the disease’s morbidity and mortality, the emergence of new strains, and the effectiveness of the vaccine. This brief report describes, evaluates, and discusses the implementation of regular antigen rapid tests (ARTs) for COVID-19 in hemodialysis units. We introduced ARTs during the surge in our hemodialysis units. As compliance with the test was mandatory by regulatory requirements, we surveyed patients and caregivers to measure their acceptability, appropriateness, and feasibility of the ART’s implementation. Acceptability measured confidence and level of comfort when performing ART tests, while appropriateness measured the perception of the necessity of ARTs, safety in the dialysis unit with the implementation of ARTs, and understanding using a Likert scale. Feasibility measured the perception of the timely start of dialysis treatment and the convenience of the test. Our survey found that ARTs were acceptable to 98% of patients and caregivers, with the majority reporting no discomfort. The majority of the patients agreed that ARTs were appropriate and feasible. We reported successful ART implementation in a healthcare setting with no false-positive or transmission within the unit during this period. Nevertheless, the long-term implementation outcome will require further evaluation.

## 1. Introduction

Parallel to the ongoing COVID-19 pandemic, there are other major present-day challenges that the global community has to wrestle with, such as climate change, political instability, Russian–Ukraine war, depletion of natural resources including food insecurity. It is crucial that we have effective strategies to contain COVID transmission and bring this epidemic to an end. The need to change habits and conduct daily living differently related to COVID has brought anxiety, stress and depression across all age groups [1].

Many countries have shifted from a ‘zero-COVID approach’ to ‘living with COVID-19’ by gradually lifting many restrictions [2]. Although vaccination programs cannot entirely prevent transmission of the COVID-19 virus, vaccination was able to reduce hospitalization, the severity of illness, and mortality [3,4]. Unlike other community settings such as schools, universities, and retail that can be converted to an online platform, physical review and contact are still required in many healthcare settings. There remain challenges in preventing transmission in many healthcare areas, especially since as many as 25% of infected patients remain asymptomatic, the occurrence of viral shedding up to 48 h before the onset of symptoms, and the constant threat of emerging variants [5,6,7]. 

Specific to end-stage renal disease (ESRD) patients in the dialysis unit, many of the potential risk drivers of transmission are unavoidable. The proximity of patients, regular contact with health care staff, prolonged treatment time and shared common facilities such as washrooms and transportation are all known risks. There are many reports of cases clustering within dialysis units [8]. Notably, our ESRD patients’ profiles are usually older with multiple co-morbidities and are at higher risk of complications and mortality if infected [9,10]. Hence, many programs took serious attention, and numerous measures have been introduced and adopted successfully in the dialysis unit to reduce the risk of transmission [5,10,11]. While specific measures, such as telemedicine, can be adopted to minimize the need for physical contact by the physician covering the dialysis unit, other healthcare staff and patients were still required to be physically present at the dialysis unit for treatment and self-isolation was not possible [12,13]. 

To reduce the risk of transmission, the Ministry of Health in Singapore (MOH) advocated for the use of self-administered antigen rapid tests (ARTs) in dialysis units. An ART is a quick screening test to triage patients who need cohorting or isolation to prevent transmission. Therefore, we designed an implementation process with strategies that is compatible with the unique characteristics of patients receiving life-sustaining dialysis in shared facilities. Following the implementation of ARTs, we conducted a survey with the aim of measuring the acceptability, appropriateness and feasibility of ARTs in the hemodialysis units. This is a first report of the adoption of pre-dialysis session ARTs and the implementation outcome.

## 2. Materials and Methods

We introduced ARTs at the end of July 2021 and started training our patients and caregivers to perform the self-administered test between 2 August 2021 and 4 August 2021 at two hemodialysis units operated by our hospital—the National University Hospital Renal Center (NUHRC) and Dialysis Center (NUHDC). To measure the implementation outcome, a cross-sectional survey was conducted six to seven weeks post implementation of mandatory ARTs. The survey was conducted between the 14 to 17 of September 2021 at NUHRC and the 20 to 22 of September 2021 at NUHDC. As implementation processes and strategies vary in different dialysis units, we performed this study in the two dialysis units operated by our hospital. Our study was conducted as part of a clinical audit and fulfilled the criteria for exemption from ethics review by the National Healthcare Group Institutional Review Board. 

### 2.1. Participants

All patients and caregivers entering the two dialysis units operated by our hospital were required to take a self-test within 24 h of each visit for dialysis treatment. A 100% compliance for ART self-testing was expected, as a negative ART result was mandatory before entry to the dialysis unit. As there was no published study measuring the implementation of ARTs, there was no minimum sample size estimated. We invited all patients and their accompanying caregivers in the dialysis units to participate. We excluded one patient with severe cognitive impairment. The survey is conducted using an online form. Self-testing is defined as the process in which a person collects his or her own nasal swab specimen, proceeds to conduct the test and interprets the results without need for professional supervision.

### 2.2. ART Kit

The ART kit we provided was the SD Biosensor Standard Q COVID-19 Ag Home Test kit approved locally by the Health Science Authority Singapore under the Pandemic Special Access Route (PSAR) [14]. ARTs are most likely to have high performance in patients with high viral loads (CT values ≤ 25), which usually appear in the presymptomatic (1–3 days before symptom onset) and early symptomatic (within the first 5–7 days of illness) phases of COVID-19 [15,16,17]. In general, ARTs can achieve a sensitivity of about 80% for cases with higher viral loads and a specificity range of 97–100% [18]. This is an easy-to-use rapid chromatographic immunoassay for the qualitative detection of SARS-CoV-2 nucleocapsid antigen present in the human nasal sample. The result of the test is ready for reading after 15 min. The process of collecting the nasal sample, mixing the sample with the prepared solution and applying the mixture to the test kit takes approximately 5 min. 

### 2.3. Implementation of ART 

Prior to introducing ARTs, we conducted brainstorming sessions to identify uptake barriers and facilitators in various patient contexts. Implementation strategies were designed to promote adoption, implementation, and sustainability. We were aware of inherent barriers to disease screening in our local population. We took into consideration convenience, cost, misconception that there is no need for screening if the person is well and without symptoms, delay in dialysis treatment start time, and fear of hospitalization if the ART was positive [19].

The implementation process was divided into 3 phases: preparation, execution, and evaluation. 

#### 2.3.1. Preparation Phase

- All patients were informed that a negative ART result was required before entry into dialysis center.
- Calculation of test kits required for patients and caregivers.

#### 2.3.2. Training Phase (Total Duration 15–20 min)

- The unit nurse conducted an in-person session with each patient, the caregiver and one family member to explain the need for enhanced screening and surveillance to minimize the risk of transmission in the unit. The session also emphasized how to interpret and respond to the ART result. The session length for each patient was approximately 5 to 10 min, although some took more time if their background health literacy was poor. The patients were advised on the following course of action:
  ○ If the ART result is negative, the patient can attend dialysis as scheduled.
  ○ If the ART result is positive, the patient is instructed to stay home and call to inform the dialysis unit. They will then be directed to report to a designated MOH center for PCR test confirmation.
  ○ If the ART result is invalid, the patient is advised to repeat the test. If the repeat test result is still invalid, the patient is to call and inform the dialysis unit.
- Patients or caregivers who are symptomatic must not present themselves to the dialysis unit and will have an arrangement made for PCR testing.
- Next, the patient and caregiver were shown a video clip demonstrating how to correctly perform the self-administered ART. The video can be viewed on https://www.youtube.com/watch?v=1-eGUzj0EsA (duration: 3 min 22 s) (accessed on 25 May 2022).
- Once the patient and caregiver have satisfactorily completed the video session and understood how self-testing with the ART kit is done, the patient and caregiver each took turns to demonstrate this under the supervision of the unit nurse in a specially assigned room. The unit nurse was gowned in appropriate Personal Protective Equipment (PPE) as recommended by the local infectious control guidelines.

#### 2.3.3. Execution Phase

- Patients and the accompanying caregivers were all instructed to do ART self-testing at home before coming to the dialysis unit for dialysis treatment. We set a validity period of 24 h before their scheduled treatment start time. Test result outside this window period could not be accepted as valid for admission into the dialysis unit.
- Most of our patients are on a conventional schedule of 3 times a week dialysis sessions. For these, ART self-testing was performed three times a week (within 24 h of each scheduled dialysis). Similarly, for patients on twice a week or four times a week or more dialysis schedule, the frequency of ART self-testing was adjusted accordingly.
- The used ART kits are returned in a disposable plastic bag with their names and personal identification numbers written on it, along with the date and time of the test. We did not accept verbal or pictorial evidence of test results. This ensured that the patient did not keep the ART kits for use in other situations or for other family members or friends. We were also mindful of the high demand for ART kits and the possibility of patients selling the kits on many of the peer-to-peer e-commerce platforms.
- Dialysis staff checked and verified the result and disposed the kits safely in the unit. The result was then documented in the hospital’s electronic medical record.
- Test kits were distributed on a “one-to-one exchange” basis.
- Frequent reminders were given on follow-up actions for positive or invalid ART results. One crucial aspect was to call from home and not come to the dialysis unit to inform the staff in person if their test results were positive or invalid as this may compromise other patients who are waiting outside the dialysis unit.

#### 2.3.4. Evaluation Phase

- Evaluation was conducted via a short online survey designed on form.gov.sg platform to monitor implementation by evaluating patients’ perception of ART for COVID-19 prevention in the dialysis unit. The online survey form is available as Supplementary Material.
- We also monitored the rate of COVID-19 acquisition and transmission in the dialysis unit.
- We reviewed the process and implementation strategy monthly and made necessary changes if needed.

### 2.4. Measuring Implementation

As there is no validated questionnaire or survey designed specifically to measure implementation outcomes of ARTs in dialysis units, we developed our questionnaire using available published survey measuring implementation outcomes [20]. Using the tabulation form, the group (all 6 authors) collectively determined the format, appropriate implementation outcome measures and length of survey to be conducted. We pretested our survey on 5 of our unit staff to check intelligibility, flow, length, and adherence as our unit staff were also subjected to routine ART self-testing 3 times a week. While there were no skipped questions, misinterpretations, or overlooked instructions, questions that took an unusually longer time to answer were modified to ensure that the questions were clearer. The revised survey was again tested before actual survey.

To measure the implementation process, we measured acceptability, appropriateness, and feasibility by conducting an online survey six weeks after the implementation and monitored COVID-19 acquisition in our dialysis units. The link to the survey was given to all patients and caregivers that accompanied the patients into the dialysis unit by scanning a Quick Response (QR) code. The implementation outcome measured the patient’s and caregiver’s perception and consisted of a total of 7 questions measuring acceptability (2 questions), appropriateness (3 questions), and feasibility (2 questions) [19]. “Acceptability” measures confidence in performing ARTs as a dichotomous variable and level of comfort with ART self-testing on an ordinal scale. “Appropriateness” involves measuring the perception of the necessity of ARTs, safety in the dialysis unit with the implementation of ARTs, and understanding the need for ARTs before each treatment, while “Feasibility” measures the perception of the timely start of dialysis treatment as a dichotomous variable and convenience of the test in Likert scale.

### 2.5. Bias

To avoid selection bias, we invited all patients and their caregivers to participate in the survey and provided translation assistance when needed. All patients were asked once to participate in the survey. Although we strongly encouraged participation, there was no coercion or undue influence in our approach, and we assured them that it was a voluntary exercise. We measured the response rate as a reflection of representativeness and degree of non-response bias. To avoid response bias, we kept the questions short and ensured that there were no leading questions. The questions were written in simple language. Likert scale was used to measure appropriateness and feasibility.

### 2.6. Outcome and Analysis

In addition to the survey, we collected data on the demographics of the respondents. Independent variables measured included the respondent’s age, gender and frequency of dialysis. Data on acceptability, appropriateness, and feasibility was presented in percentage or by the distribution of responses. A positive response was measured as agree and strongly agree on the Likert scale. Because the aim of the study was descriptive, results are presented without any forms of statistical inference. No exploratory statistical tests were performed due to the small sample size.

## 3. Results

NUHRC is the only hospital-based outpatient facility in Singapore, set up specifically as a step-down unit for higher medical dependency ESRD patients, while NUHDC is a satellite facility that dialyzes more stable ESRD patients off-site. NUHRC has eight dialysis stations and performs treatment for 23 patients, while NUHDC has 12 dialysis stations and performs treatment for 57 patients. There was a total of 80 patients and 38 caregivers in the two dialysis units during the survey period.

A total of 2255 ART self-testing results were recorded from the time ARTs were implemented to the time of the survey. A total of 463 and 1792 ARTs were performed at NUHRC and NUHDC, respectively. We achieved a response rate of 83.7 % for the online survey.

The demographic data of survey respondents are reported in Table 1. This was a cross-sectional survey, and each patient and caregiver were invited to complete the survey only once. We did not record any cases of positive ARTs during the period when mandatory ARTs were first adopted until the time this survey was conducted. There were also no cases of confirmed COVID-19 from these 2 units during that period of time.

Our study found that self-administered ARTs were acceptable, with 98% of respondents indicating confidence in performing ARTs independently. Most (96%) felt no or mild discomfort in performing the test. We received positive responses in all domains of appropriateness, with most respondents agreeing that the implementation of ARTs was necessary. They also felt subjectively safer and understood the need for compliance with COVID-19 measures in the dialysis unit, even if they had a negative ART (Table 2). In terms of feasibility, most respondents agreed that ARTs were convenient (84%) and that dialysis was started without delay (100%).

Specific to acceptability, the patients who had no confidence and had moderate discomfort were patients in the older age group (>60 years). For appropriateness, interestingly, respondents who felt ARTs were not necessary prior to dialysis somehow agreed that the dialysis unit was safer knowing all other patients and caregivers were tested before entering the unit. Overall, there was no observable demographics pattern or association among patients that reported ARTs positively and those that did not. As the numbers were low, no statistical test was performed.

## 4. Discussion

Since the onset of the pandemic, we have adopted various infection control measures at the dialysis unit [5,21]. MOH initiated compulsory ARTs on 7 August 2021 with aim to use a low cost easily scalable point-of-care test to detect asymptomatic COVID-19 cases before entry into dialysis units to reduce the risk of transmission [22,23]. We adopted the fundamental of implementation science to offer a systematic approach to design, execute and then measure the acceptability, appropriateness, and feasibility of administering self-testing ARTs before each dialysis session. Our result shows that the whole process, from design to satisfactory execution, can be achieved without needing extensive time and effort. 98% of the respondents found it acceptable and easy to perform; 96% reported no or little discomfort performing ART testing. The respondents all responded positively to the survey questions on appropriateness. The majority agree or strongly agree that ARTs are a necessary screening tool before each dialysis as they will enhance safety in the dialysis unit. Over 80% reported that it was convenient, and no patients were delayed in starting dialysis treatment as the ART testing was carried out at home.

World Health Organization (WHO) and the Centers for Disease Control and Prevention (CDC) have recommended reverse-transcription polymerase chain reaction (RT-PCR) as the standard diagnostic assay for SARS-CoV-2 detection RT-PCR has a high sensitivity for SARS-CoV-2, ranging from 71 to 98%, and the assay was 100% specific [24,25]. However, PCR tests require a trained person to perform the test, laboratory capacity, costly reagents, and long processing time. This often causes delays and barriers to the patient needing treatment, such as in a dialysis unit when there is time pressure to complete the treatment and a high turnover of patients [26]. Although it is well established that sensitivity is higher in symptomatic persons with higher viral load, reported sensitivity of the ART kits was 95.7% at the minimal viral load compatible with contagiousness [27]. This is reassuring, given that we will be able to detect more than 95% of those who are infectious, and the remaining false negatives are those with a much lower risk of being infectious [27]. Uniquely, repeated testing 3 times a week, as mandated in a dialysis unit, will further reduce the risk of a false negative result. Not only do ARTs have high sensitivity and excellent specificity in detecting SARS-CoV-2 in individuals in the community, compliance to ARTs has also been reported to be high among other areas in the community [28,29].

The role of ARTs has gradually evolved as most countries are managing the disease as endemic in their population. ART screening for caregivers enabled patients who may be dependent (including those with cognitive impairment or impaired mobility) to be accompanied during their dialysis sessions [30]. This allows for care continuity and avoid excessive taxation on nursing manpower. There has been only one published study on ART self-testing in the hemodialysis units. A group in Italy conducted a retrospective evaluation of a routine universal bimonthly ART performed for all in-center hemodialysis patients without symptoms. [31] A total of 4079 ARTs were performed on 277 patients. There were 38 positive ARTs, but only 5 were RT-PCR positive. As it was not a prospectively designed study, ART negative patients were not tested by RT-PCR unless the patient became symptomatic or had other clinical indications. The authors reported that 219 patients did have symptoms or clinical indications for RT-PCR during that period, and 13 more patients were diagnosed with COVID-19 infection. They concluded that a bimonthly ART schedule was perhaps not very useful for the early detection of COVID-19 patients in a dialysis unit because of the short incubation period of this disease. Hence, a 3 times a week (or a twice a week) ART schedule is recommended if attendance is recurrent and necessary over short intervals, as in a dialysis unit.

Implementation outcomes provide a means to evaluate the implementation success of a given, usually evidence-based, intervention (such as screening test, treatment, and bundled protocol) and are distinct from other, traditionally measured outcomes. Proctor and his colleagues developed eight distinct components as a framework to measure and conceptualize implementation outcomes [32]. These are acceptability, adoption, appropriateness, cost, feasibility, fidelity, penetration, and sustainability [33]. Each of these unique characteristics will influence and determine the successful eventual implementation of the desired intervention. As ARTs were a new but mandatory screening protocol prior to attending each dialysis session in the dialysis facility, it was important for us to measure acceptability, appropriateness, and feasibility, as these three patient factors will contribute to the sustainability of the program. MOH and our hospital have already included measures that will overcome the challenges of the remaining components. Adoption and penetration are no longer barriers to implementation as patients are told that a negative ART result is a prerequisite before they can be considered for treatment in these outpatient facilities. The cost factor is also removed as the hospital provided free test kits for all patients and one caregiver (if needed). The fidelity of the test kit used is confirmed by the hospital providing the ART kits. To reduce financial barriers to healthcare and to encourage appropriate care-seeking behaviors during a pandemic, the cost of screening and other infection control measures such as vaccination as well as hospitalization related to COVID-19 were fully financed by the government of Singapore. Countries with different healthcare financing system and healthcare governance structure will need to resolve implementation issues and make it compatible with their local healthcare landscape.

The deployment of ARTs as a screening test in community and healthcare settings is dependent on the trend of new cases, severity of those infected, population vaccination rate, and how the public health officers and the government balance infection control measures with the medical and socio-economic consequences of those measures. Countries with stable and acceptable community transmission rates may transit to live with COVID-19 as an endemic disease in the population and scale down the intensity of screening and other public health measures, especially in low-risk settings. However, not every country is ready to take this approach. Some may still be experiencing a steep rise in daily cases and for various reasons have a low vaccination rate. ART was a useful tool for the workplace, school, nursing homes, dental clinics, clinics that perform aerosol generating procedures and hospital emergency rooms at the height of the pandemic in many countries [33,34,35]. In addition, ART may be particularly useful in childcare and schools. Asymptomatic infection is common in children and vaccination rate for children may be low as authorization for COVID vaccine use in children was only granted later. In a 2020 systematic review of 18 reviews of symptoms and signs in persons <20 years of age with documented COVID-19 infection, the proportion of asymptomatic infections ranged from 15 to 42 percent [36]. In a subsequent review of electronic health records of 82,798 United States children <18 years of age with laboratory-confirmed COVID-19 infection between March 2020 and December 2021, 66 percent were asymptomatic [37].

The primary limitation of the study was that this is a single-center experience conducted in a Singapore dialysis population involving relatively small number of respondents. The validation index of the survey was not measured due to limited time and resources during the period of COVID-19 surge. Socio-cultural background and ethnicity of the population being surveyed may yield different outcomes. We do acknowledge this and have taken the effort to conduct this survey in an anonymous manner to reduce any potential bias. Cost factor may also influence a person’s view on their willingness to adopt a recommended measure and their perception of necessity. This is not a factor in our context as the ART kits are provided without cost to patients and their caregivers.

The strength of this study is that it is the first to examine the routine implementation of ARTs in two outpatient dialysis units, one hospital-based and one satellite unit. Our implementation strategies were acceptable, appropriate, and feasible. We demonstrated one means of ensuring continuity of essential care without compromising the quality of care or adding burden to our patients, who are primarily elderly with multiple comorbidities. From a universal public health standpoint, dialysis units outside of Singapore may consider adopting this strategy of COVID-19 risk mitigation in their unit. Other healthcare settings that may consider it necessary or advantageous to require ART self-testing as part of the workflow can broadly adapt our strategies and make targeted modifications as needed to suit the unit’s characteristics and circumstances. Implementation science framework can be practical across different sets of challenges and requirements. Different implementation outcomes may be selected in accordance with the local environment and, crucially, should be measured.

## 5. Conclusions

In conclusion, the implementation of ART self-testing in the outpatient dialysis unit was acceptable, appropriate, and feasible for patients according to the result of this survey. The patients and caregivers reported a positive experience with the test. The emergence of COVID-19 has brought major changes in human behaviors, institutions and societies. For any new proposed COVID-19 measures that affect the patients and require their full participation, implementation science is an approach that is systematic and confers a higher chance of successful adoption and execution. Measuring implementation outcomes will serve as a supporting process that provides insights to what will make it sustainable.

## Figures and Tables

**Table 1:** Demographic data of survey respondents.

	Total (*n* = 98)	NUH RC (*n* = 19)	NUHDC (*n* = 79)
Respondent, *n* (%)			
Patient	62 (63.3)	13 (68.4)	49 (62.0)
Care Giver	36 (36.7)	6 (31.6)	30 (38.0)
Gender, *n* (%)			
Female	51 (52.0)	7 (36.8)	44 (56.0)
Male	47 (48.0)	12 (63.2)	35 (44.0)
Age Group, *n* (%)			
<30	5 (5.1)	1 (5.3)	4
30–59	33 (33.6)	9 (31.0)	24
>60	60 (61.2)	9 (31.0)	51
ARTs taken per week, *n* (%)			
2 times/week	5 (5.1)	2 (10.5)	3 (3.8)
3 times/week	80 (81.6)	11 (57.9)	69 (87.3)
4 times/week	12 (12.2)	6 (31.6)	6 (7.6)
Others	1 (1.0)	0 (0)	1 (1.3)

**Table 2:** Appropriateness of ARTs.

Measurement	Results
Appropriateness (3 questions)	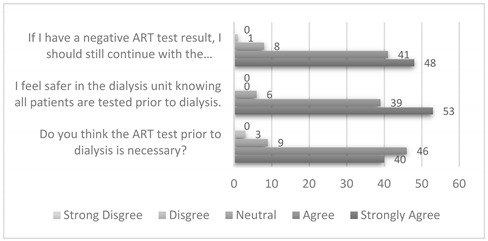

## Data Availability

The data presented in this study are available on request from the corresponding author with data sharing agreement.

## Supplementary Materials

The following supporting information can be downloaded at: https://www.mdpi.com/article/10.3390/ijerph192215319/s1.
