# Supplementary material for: The Implementation and Role of Antigen Rapid Test for COVID-19 in Hemodialysis Units
Source: Int J Environ Res Public Health. 2022 Nov 19;19(22):15319. doi: 10.3390/ijerph192215319 (PMC9690273; doi:10.3390/ijerph192215319)
Supplement: Supplementary file 1 [file ijerph-19-15319-s001.zip › ijerph-1918406-supplementary.pdf]

Survey

Dear patients and caregivers,

We would like to invite you to participate in an online survey.

The hemodialysis team at National University Hospital is conducting this survey to better understand your views about ART (Antigen Rapid Test) implementation in our units.

The survey is very brief and will not take longer than 10 minutes to complete. Please click on the link below to proceed with the survey.

Your participation in this survey is voluntary and responses are strictly anonymous.

By completing this survey, you are helping us to better design services for our dialysis patients.

Thank you

Sabrina Haroon Wong MD

1. Your status
□ Patient
□ Cargiver

2. Your gender
□ Male
□ Female

3. Your age group
□ < 30 years
□ 30-59 years
□ ≥ 60 years

4. Frequency of dialysis
□ 2 times/week
□ 3 times/week
□ 4 times/week
□ Other

5. Do you feel confident performing ART?
□ Yes
□ No

6. Are there any discomfort while performing ART at home?
□ No discomfort
□ Mild discomfort
□ Moderate discomfort
□ Significant discomfort

7. Self-administered ART prior to each dialysis is necessary.
□ Strongly agree
□ Agree
□ Neutral

□ Disagree
□ Strong disagree

8. I feel safer in the dialysis unit knowing all the patients are tested prior to dialysis.
□ Strongly agree
□ Agree
□ Neutral
□ Disagree
□ Strong disagree

9. If I have a negative ART test result, I should still continue with the precautionary measures while I am in the dialysis unit.
□ Strongly agree
□ Agree
□ Neutral
□ Disagree
□ Strong disagree

10. Was your dialysis therapy delayed because of the need to verify ART result?
□ Yes
□ No

11. Self-administered ART prior to dialysis is a convenient method of COVID-19 screening.
□ Strongly agree
□ Agree
□ Neutral
□ Disagree
□ Strong disagree
